# High levels of HIV drug resistance among adults failing second-line antiretroviral therapy in Namibia

**DOI:** 10.1097/MD.0000000000021661

**Published:** 2020-09-11

**Authors:** Michael R. Jordan, Ndapewa Hamunime, Leonard Bikinesi, Souleymane Sawadogo, Simon Agolory, Andreas N. Shiningavamwe, Taffa Negussie, Christa L. Fisher-Walker, Elliot G. Raizes, Nicholus Mutenda, Christian J. Hunter, Natalie Dean, Kim Steegen, Vibha Kana, Sergio Carmona, Chunfu Yang, Alice M. Tang, Neil Parkin, Steven Y. Hong

**Affiliations:** a Division of Geographic Medicine and Infectious Disease, Tufts Medical Center; b Department of Public Health and Community Medicine, Tufts University School of Medicine, Boston, MA, USA; c Directorate of Special Programmes, Ministry of Health and Social Services, Windhoek, Namibia; d Parsyl, Inc., Denver, CO, USA; e United States Centers for Disease Control and Prevention; f Namibia Institute of Pathology; g Department of Internal Medicine, University of Namibia, Windhoek, Namibia; h Department of Biostatistics, University of Florida, Gainesville, FL, USA; i Department of Molecular Medicine and Haematology, University of Witwatersrand, Johannesburg, South Africa; j United States Centers for Disease Control and Prevention, Atlanta, GA; k Data First Consulting, Inc., Sebastopol, CA, USA.

**Keywords:** HIV drug resistance, Namibia, protease inhibitor, sub-Saharan Africa

## Abstract

To support optimal third-line antiretroviral therapy (ART) selection in Namibia, we investigated the prevalence of HIV drug resistance (HIVDR) at time of failure of second-line ART. A cross-sectional study was conducted between August 2016 and February 2017. HIV-infected people ≥15 years of age with confirmed virological failure while receiving ritonavir-boosted protease inhibitor (PI/r)-based second-line ART were identified at 15 high-volume ART clinics representing over >70% of the total population receiving second-line ART. HIVDR genotyping of dried blood spots obtained from these individuals was performed using standard population sequencing methods. The Stanford HIVDR algorithm was used to identify sequences with predicted resistance; genotypic susceptibility scores for potential third-line regimens were calculated. Two hundred thirty-eight individuals were enrolled; 57.6% were female. The median age and duration on PI/r-based ART at time of enrolment were 37 years and 3.46 years, respectively. 97.5% received lopinavir/ritonavir-based regimens. The prevalence of nucleoside reverse transcriptase inhibitor (NRTI), non-nucleoside reverse transcriptase inhibitor (NNRTI), and PI/r resistance was 50.6%, 63.1%, and 13.1%, respectively. No significant association was observed between HIVDR prevalence and age or sex. This study demonstrates high levels of NRTI and NNRTI resistance and moderate levels of PI resistance in people receiving PI/r-based second-line ART in Namibia. Findings underscore the need for objective and inexpensive measures of adherence to identify those in need of intensive adherence counselling, routine viral load monitoring to promptly detect virological failure, and HIVDR genotyping to optimize selection of third-line drugs in Namibia.

## 1 Introduction

As of July 2017, 59% of all people living with HIV worldwide were receiving antiretroviral therapy (ART).^[1]^ Increased access to antiretroviral (ARV) drugs has led to increasing levels of drug resistant HIV.^[2,3]^ International guidelines recommend ritonavir-boosted protease inhibitor (PI/r)-based ART as an effective second-line strategy after failure of non-nucleoside reverse transcriptase (NNRTI)-based first-line ART.^[4]^

Global HIV drug resistance (HIVDR) surveillance efforts and the majority of studies performed in low- and middle-income countries have focused on estimating the prevalence of HIVDR prior to treatment initiation or after failure of first-line NNRTI-based therapies.^[2]^ Thus, comparably less information is available to guide selection of optimal regimens in people with virological failure while taking second-line ART.

ART in Namibia is delivered following a public health approach, which involves use of standardized first- and second-line regimens and simplified laboratory monitoring, including at least one viral load test per year. At the time of study enrolment, Namibia's national ART guidelines recommended first-line regimens consisting of two nucleoside reverse transcriptase inhibitors (NRTI), tenofovir (TDF)/emtricitibine (FTC) or lamividuine (3TC) administered with the NNRTI efavirenz (EFV). Recommended second-line regimens were three NRTI (3TC or FTC, TDF, zidovudine (ZDV), or abacavir (ABC)) administered with a ritonavir-boosted protease inhibitor, either lopinavir/ritonavir (LPV/r) or atazanavir/ritonavir (ATV/r).

At time of study initiation (2016), 140,241 adults out of an estimated 204,147 adults living with HIV were receiving ART in Namibia of which 3884 were taking PI/r-based second-line regimens (unpublished data, Namibia Ministry of Health and Social Services). Globally and in Namibia, unanswered questions remain about the contribution of protease inhibitor (PI) drug resistance to second-line ART failure. In this study, we report the prevalence and patterns of HIVDR in people failing second-line ART in Namibia's public health ART program.

## 2 Methods

### 2.1 Study design

The 15 ART clinics with the largest number of people on PI/r-based ART in the country were selected. These 15 clinics captured 70% (2746 of 3884) of all people receiving PI/r-based ART; clinics were located in nine different geographical regions: Khomas, Ohangwena, Zambezi, Oshikoto, Oshana, Kavango East, Erongo, Omusati, and Otjozondjupa.

Between August 2016 and February 2017, all HIV-infected people ≥15 years of age receiving second-line PI-based ART for at least six months and who had confirmed virological failure per Namibia national ART guidelines (two consecutive HIV RNA tests ≥1000 copies/mL separated by at least three months) were identified and asked to participate in the study during routine clinic visits. Written informed consent was obtained from all participants. Individuals with self-reported treatment interruption of 30 days or more at the time of enrolment were ineligible; no additional information was collected about this group.

### 2.2 Data and specimen collection and HIVDR sequencing

At study sites, nurses drew 5 mL of whole blood via venepuncture for viral load (VL) testing (a third consecutive VL test). Whole blood specimens were transported in a cold box on the day of collection to the Namibian Institute of Pathology (NIP) for VL testing. On arrival at NIP, dried blood spot (DBS) specimens were prepared by pipetting 75 μL aliquots of whole blood on each of 5 circles of a Whatman 903 filter paper card (GE Healthcare, Marlborough MA, USA). DBS were dried at room temperature for 24 hours and subsequently packed in individual gas impermeable bags per World Health Organization (WHO) guidance^[5]^ and were frozen at –80^o^C for storage. DBS from participants who had a third consecutive VL test ≥1000 copies/mL were brought to ambient temperature, re-packed into new gas impermeable bags with desiccant and humidity indicator cards and shipped to South Africa for HIVDR testing.

VL testing was performed on plasma within 48 hours of specimen receipt using COBAS AmpliPrep/COBAS TaqMan HIV-1 Test (Roche Molecular Systems, Inc., Branchburg, NJ, USA).

HIV-1 protease and reverse transcriptase (RT) sequences were obtained using standard population sequencing adapted from previously described methods.^[6]^ HIVDR testing was performed at the National Health Services Laboratory, University of Witwatersrand, Johannesburg, South Africa, a WHO-designated HIVDR genotyping laboratory. RECall^[7]^ was used to generate consensus sequences; quality assurance was performed following WHO recommendations.^[6]^ The Stanford HIV drug resistance algorithm (HIVdb), version 8.4, was used to identify HIVDR resistance profiles, mutations, and HIV subtypes^[8]^; sequences classified as having low-, intermediate-, or high-level HIVDR were considered as resistant. The HIV-1 integrase region was not sequenced because at the time of this study integrase strand transfer inhibitors (InSTI) were not prescribed for treatment of HIV in Namibia's public health sector.

Potential third-line treatment options were considered based on an algorithm developed by South Africa's national third-line ART committee.^[9,10]^ The algorithm was used becasue Namibia does not have an established algorithm for selection of third-line ART based on HIVDR genotype test results. Genotypic susceptibility scores (GSS) for individuals who would have been eligible for third-line ART (i.e., those with predicted resistance to the PI/r included in their second-line regimen) were calculated based on HIVdb penalty scores for each drug in the regimen that would have been prescribed following the algorithm. The ARV drugs used in the algorithm include darunavir/ritonavir (DRV/r), 3TC/FTC, ZDV or TDF (whichever has the lowest penalty score), and an InSTI if the TDF or ZDV score is 30 or above or the DRV/r score is 15 or above. In addition, etravirine (ETR) is added if the TDF or ZDV score is 30 or above and the DRV/r score is 15 or above and the ETR score is below 30. One GSS point was given for each drug with a Stanford HIVdb penalty score <15; it was assumed that virus was fully susceptible to all approved InSTI.

### 2.3 Statistical analysis

Analyses were performed using Stata (version 15.1; College Station, TX, USA).^[11]^ Demographic and laboratory characteristics are summarized as prevalence values or median values and interquartile ranges (IQR). HIVDR prevalence is reported with 95% Wilson confidence intervals adjusting for site-level clustering using a robust standard error. Fisher's exact tests and Wilcoxon rank sum tests were used to conduct two-group comparisons with a significance level of 0.05.

### 2.4 Ethical statement

The study received institutional review board (IRB) approval from the Ministry of Health and Social Services in Namibia and expedited review from the IRBs of Tufts University School of Medicine, the University of Witwatersrand, Johannesburg, South Africa. The study was reviewed in accordance with the Associate Director for Science at the Center for Global Health, United States Centers for Disease Control and Prevention CDC human research protection procedures and was determined to be research. CDC investigators did not interact with human subjects or have access to identifiable data or specimens for research purposes.

## 3 Results

All 238 eligible individuals identified during the study period were enrolled; 96.6% (230/238) were receiving a regimen consisting of three NRTIs in combination with either ATV/r or LPV/r. Table 1. At time of second-line ART initiation, median CD4 cell count was 241 cells/μL (IQR 137,447) and median time from second-line treatment initiation to confirmed virological failure (programmatically defined as participant's second consecutive VL ≥1000 copies/mL on PI/r-based regimen) was 2.22 years (IRQ 1.22, 3.69). Median times from first documented virological non-suppression (first VL > 1000 copies/mL) to confirmed virological failure and from initiation of second-line treatment initiation to study enrolment were 0.69 years (IRQ 0.37, 1.7) and 3.46 years (IQR 1.86, 5.16), respectively.

**Table 1:**
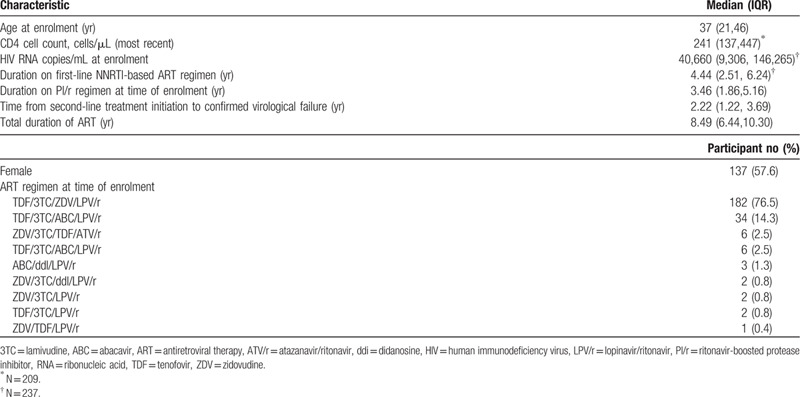
Participant characteristics at study enrolment in Namibia (n = 238).

HIVDR genotyping was successful for 160/238 (67.2%) participants. Overall 70.0% (112/160) of sequences had mutations conferring drug resistance to any drug; 50.6%, 63.1%, 13.1%, and 6.9% had any NRTI, any NNRTI, LPV/r or ATV/r, DRV/r resistance, respectively (Table 2). No clinically relevant resistance was predicted in 48 (30.0%) participants. No significant differences were observed with respect to sex, age, and median VL at time of confirmed virological failure between people with and without genotypes available for analysis (data not shown). HIV-1 subtype C predominated (n = 154; 96.3%), followed by CRF02_AG (n = 3; 1.9%), HIV-1 G (n = 2; 1.3%), and CRF45_cpx (n = 1; 0.6%).

**Table 2:**
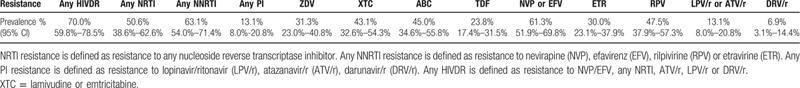
Prevalence of HIV drug resistance amongst second-line ART failures in Namibia (n = 160).

**Figure 1:**
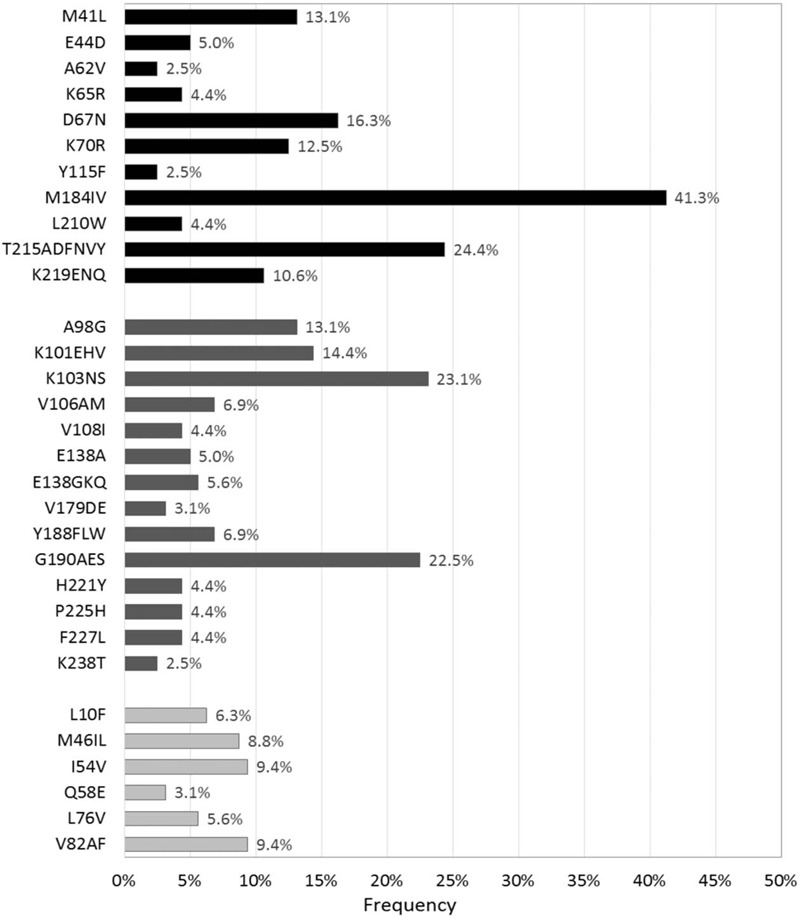
Frequency of HIV drug resistance mutations detected greater than 2% of all sequences. Non-nucleoside reverse transcriptase inhibitor mutations are shown in black; nucleoside reverse transcriptase inhibitor mutations are shown in dark gray; protease inhibitor mutations are shown in light gray.

The frequencies of RT inhibitor and PI mutations detected in over 2% of all sequences analysed are presented in Figure 1. M184V (41.3%) was the most frequently observed NRTI mutation followed by T215A, F, D, V, N, or Y (24.4%) and D67N (16.3%). K70R was observed in 12.5% and K65R in only 4.4%. The most frequently observed NNRTI mutation was K103N or S (23.1%) followed by G190A, E, or S (22.5%), K101E, H, or V (14.4%), and A98G (13.1%). I54V (9.4%) and V82A or F (9.4%) were the most commonly occurring PI mutations followed by M46I or L (8.8%). The major PI mutation L90M occurred at a frequency of 1.3% of all sequences analysed. 18 of the 21 (85.7%) sequences with major PI drug resistance mutations also had M184I or V.

Although overall PI resistance was modest, of those with PI resistance, 71.4% (15/21) had three or more major PI mutations (Table 3). In addition, of those with PI resistance, 20 (95.2%), 19 (90.5%), 19 (90.5%), and 18 (85.7%) had resistance to ZDV, TDF, ABC, and 3TC or FTC, respectively. No significant association was observed between HIVDR prevalence and age (≥25 years vs < 25 years, 64.3% vs 73.1%) or sex (female vs male, 72.2% vs 66.7%).

**Table 3:**
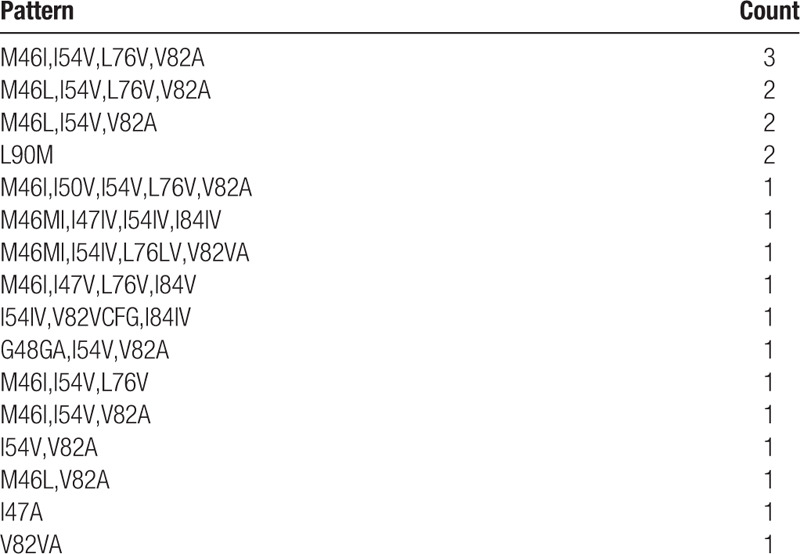
Protease inhibitor mutational profiles in individuals with protease inhibitor resistance in Namibia (n = 21).

GSS scores for potential third-line regimens following guidance developed by South Africa's national third-line ART committee^[10,11]^ are presented in Table 4. Twenty one individuals would have been deemed eligible for third-line ART by virtue of having virus with predicted resistance to the PI component of their second-line regimen (LPV/r or ATV/r). Of these, 62% (13/21) would have received a regimen with a GSS score of 1, with 6 of the 13 having virus susceptible to DRV/r only and 7 susceptible to the InSTI only. Eight individuals (38%) would have received a regimen with a GSS score of 2 or 3.

**Table 4:**
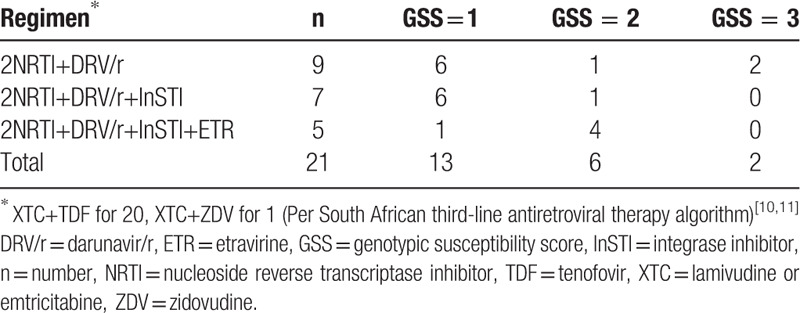
Genotypic susceptibility scores for possible third-line ART regimens.

## 4 Discussion

This study of HIVDR in people failing PI/r-based second-line ART in Namibia has important findings. First, 30% of people failing PI/r-based ART had no predicted HIVDR and only 13% had PI resistance, suggesting that most virological failures were not due to drug resistant virus. This observation suggests that greatly intensified adherence support may be an optimal initial approach to patient management of viral non-suppression in people receiving PI/r ART in Namibia. Secondly, our findings support the importance of routine VL monitoring for early detection of suboptimal adherence, followed by HIVDR testing in individuals identified as failing therapy, an approach which is likely to minimize premature switch to more complex and costly third-line regimens.

In this study, overall NRTI resistance levels were high and could lead to reduced efficacy of the NRTI backbone for half of the people evaluated for third-line PI/r-based regimens. However, the comparably low prevalence of PI resistance observed in this study is reassuring given that genotypic testing may not accurately predict regimen activity when providing PI/r-based ART after second-line PI failure: PI/r-based regimens have demonstrated efficacy in people with NRTI resistance, even when predicted NRTI resistance is complete, suggesting that NRTIs retain partial antiretroviral activity or that NRTI-resistant viruses have reduced fitness.^[12–14]^

Thirteen per cent of study participants failed with PI-resistant virus, emphasizing the need for HIVDR testing in people receiving PI/r-based second-line treatment to ensure the selection of highly potent third-line regimens (including use of InSTI with optimal NRTI backbones) and to identify individuals with virological failure due to suboptimal adherence (i.e., those with no PI/r resistance) who are likely to benefit from enhanced adherence counselling. Although 87% of patients in this cohort have no PI resistance, informal patient surveys in Namibia's national ART programme suggest that the majority of people failing PI/r-based ART report inconsistent adherence to the PI component of the regimen due to PI-specific toxicity or side effects; thus resulting in dual therapy (personal communication, Dr. Leonard Bikinesi, Chief Clinical Mentor HIV Programme, Directorate of Special Programmes, Ministry of Health and Social Services, Windhoek, Namibia). Because of expense and logistics, HIVDR genotyping remains a costly adherence test, presenting an additional challenge: the lack of robust and inexpensive objective measures adherence to ART and specifically the PI/r component, which may lead to delayed switch in the face of true HIVDR or premature switch in people with no HIVDR.

The prevalence estimates of overall, drug class and drug-specific resistance observed in this study are broadly comparable to those observed in other countries^[15–17]^ and in a recent retrospective non-representative convenience sample of 366 HIVDR genotypes obtained by Namibia's ART program during the period 2010 to 2015.^[18]^

The predicted activity of potential third-line regimens according to South Africa's national algorithm is somewhat encouraging, since most (14/21, 67%) patients were either susceptible to DRV/r or had at least two active drugs available. A recent publication assessing the efficacy of South Africa's third-line algorithm showed a high proportion of individuals on third-line had achieved HIV viral suppression (83%, VL <1000 copies/mL) after a minimum of six months^[11]^ In addition, in a recent meta-analysis, DRV/r monotherapy in ART-experienced people was shown to be effective.^[19]^ However, one-third (7 of 21) of people in this Namibian cohort would have been treated with regimens having suboptimal predicted efficacy because their viruses were fully susceptible to the InSTI only. Despite its low propensity to select for drug resistance mutations, there are an increasing number of reports of virological failure associated with InSTI resistance in ARV drug-experienced InSTI-naïve individuals receiving a dolutegravir-containing regimen,^[20,21]^ underscoring the need for caution and close virological monitoring of this sub-population who may be at risk of virological non-suppression.

Medication non-adherence appears to be an important cause of second-line ART failure in one third of people in this study. A limitation of this study is that pharmacy refill data, pill count, or plasma, hair, or urine drug levels were unavailable; therefore, objective measures of adherence could not be assessed. This study has additional limitations. Although study sites comprised over 70% of all people receiving PI/r in Namibia at the time of the study, sites may have differed from other regions in the country with respect to patient demographics or ARV drug treatment histories by virtue of their larger patient populations. Although amplification success rates were similar across all clinics, there is a possibility that specimens that failed to amplify may have more or less drug resistance than those that amplified; however, as described, there was no differences between people with and without genotypes available for analysis. In addition, nearly all study participants were receiving three NRTIs in addition to PI/r (as per Namibia guidelines^[22]^; therefore these results may not be generalizable to populations failing WHO-recommended second-line ART regimens consisting of only two NRTIs and PI/r. Nonetheless, our findings reflect the majority of those adults failing second-line ART in Namibia and provide important information that can be used programmatically for the management of people experiencing virological failure of PI/r-containing regimens in the country.

## 5 Conclusions

This study demonstrates high levels of NRTI resistance and moderate levels of PI resistance in people receiving PI/r-based second-line ART, supporting the need to optimizing treatment adherence to all three second-line ARV drugs, use of routine VL monitoring to promptly detect virological failure, and HIVDR genotyping to optimize selection of third-line drugs. In addition, programmatic concerns about selective non-adherence to PI/r argue for more tolerable highly potent and fixed-dose combination regimens.

## Author contributions

MRJ and SYH conceived the study. MRJ wrote the protocol, designed the study and wrote the first draft of the manuscript. NH, LB, SS, SA, ANS, TN, CLF-W, EGR, NM, CJH, and AMT supported implementation of the study and interpretation of data in light of Namibia's public health ART Programme. ND performed statistical analysis. KS, VK, SC perofmred HIVDR genotyping and quality assurance of sequences. MRJ and NP performed additional sequence quality assurance and performed the HIVDR interpretation and genotype susceptibility scoring. All authors contributed to the final draft of the manuscript and had access to the data.
